# Vorschlag für ein Verfahren zur Teilnahme an intensiv- und notfallmedizinischen Studien bei nichteinwilligungsfähigen Patient*innen (Kölner Modell)

**DOI:** 10.1007/s00063-023-01063-2

**Published:** 2023-09-29

**Authors:** M. Kochanek, G. Grass, B. Böll, D. A. Eichenauer, A. Shimabukuro-Vornhagen, M. Hallek, T. Zander, J. Mertens, R. Voltz

**Affiliations:** 1 Medizinische Fakultät und Universitätsklinik Köln, Med. Klinik I für Innere Medizin, Center for Integrated Oncology Aachen Bonn Cologne Düsseldorf (CIO), Universität zu Köln, Kerpener Str. 62, 50937 Köln, Deutschland; 2 Ethikkommission der Medizinischen Fakultät, Universität zu Köln, Köln, Deutschland; 3 Amtsgericht Köln, Köln, Deutschland

**Keywords:** Klinische Prüfung, Ethikkommission, Ehegattenvertretungsrecht, Betreuung, Einwilligungserklärung, Clinical trials, Ethics committee, Spousal representation law, Guardianship, Informed consent

## Abstract

Bei der Durchführung von klinischen Studien in der Intensiv- und Notfallmedizin bestehen bei nichteinwilligungsfähigen Patient*innen unterschiedliche Ansichten von Mediziner*innen, Ethikkommissionen und Jurist*innen. Durch differente Ansichten über die Teilnahme von nichteinwilligungsfähigen Patient*innen wird damit zusätzlich die Vorbereitung und Durchführung von klinischen Prüfungen erschwert. Mittels entsprechender Literaturrecherche konnte ein Konsensmodell (Kölner Modell) durch klinisch forschende Ärzt*innen, Ethikkommission und Jurist*innen erstellt werden, dass sowohl Patient*innen, den für die Studie wissenschaftlich Verantwortlichen als auch Ethikkommissionen und Betreuungsrichter*innen ein Höchstmaß an Patient*innensicherheit sowie Rechtssicherheit bringt und gleichzeitig wissenschaftliche Forschung ermöglicht.

## Abstract (en)

When conducting clinical trials in intensive care and emergency medicine, physicians, ethics committees, and legal experts have differing views regarding the inclusion of patients who are incapable of giving consent. These different views on the participation of patients who are not capable of giving consent also complicate how clinical trials are prepared and conducted. Based on the results of a literature search, a consensus model (Cologne Model) was developed by physicians performing clinical research, ethics committees, and lawyers in order to provide patients, those scientifically responsible for the study, ethics committees, and probate (guardianship) judges with a maximum of patient safety and legal certainty, while simultaneously enabling scientific research.

## Einleitung

In den letzten Jahren hat die Forschung im intensiv- und notfallmedizinischen Bereich stark zugenommen. Der großen Bedeutung der Forschung in der Intensiv- und Notfallmedizin stehen ethische Bedenken gegenüber, Studien bei nichteinwilligungsfähigen Patient*innen durchzuführen. In Deutschland bestehen von Mediziner*innen, Ethikkommissionen und juristischer Seite unterschiedliche Ansichten zu Anforderungen im Hinblick auf die Teilnahme von nichteinwilligungsfähigen Patient*innen an wissenschaftlichen Studien. Dies kann etwa bei multizentrischen Studien in einzelnen teilnehmenden Zentren zu Irritationen führen.

Mit Wirksamwerden der Verordnung (VO) (EU) Nr. 536/2014 des Europäischen Parlaments und des Rats vom 16. April 2014 über klinische Prüfungen mit Humanarzneimitteln und zur Aufhebung der Richtlinie 2001/20/EG (im Folgenden: VO (EU) Nr. 536/2014) werden die Rechtsvorschriften für die Durchführung klinischer Prüfungen in Europa harmonisiert [1]. Diese Rechtsverordnung ist am 31.01.2022 wirksam geworden mit einer Übergangszeit bis zum 30.01.2025. Ein Novum dieser Verordnung ist die nicht mehr ortsgebundene Bewertung der klinischen Prüfung durch die für die Leitung der klinischen Prüfung zuständigen lokalen Ethikkommission, sondern durch eine gemäß Geschäftsverteilungsplan zugeordnete Ethikkommission. Dadurch können differente Ansichten über die Teilnahme von nichteinwilligungsfähigen Patient*innen im intensiv- und notfallmedizinischen Bereich zusätzlich die Vorbereitung und Durchführung von klinischen Prüfungen erschweren. Ziel war es daher, ein Modell zu entwickeln, das sowohl die Patient*innen und den für die Studie wissenschaftlich Verantwortlichen als auch Ethikkommissionen und Betreuungsrichter*innen ein Höchstmaß an Patient*innensicherheit sowie Rechtssicherheit bringt und gleichzeitig wissenschaftliche Forschung ermöglicht. Es kann damit auch ein zusätzliches Instrument in der europäischen Harmonisierung zur Durchführung von klinischen Studien sein.

## Methode

Es wurde eine ausführliche Literaturrecherche zum Thema Aufklärung von nichteinwilligungsfähigen Patient*innen für intensiv- und notfallmedizinische Studien durchgeführt. Für die Literaturrecherche wurden nur Publikationen mit dem Thema Intensivmedizin, Notfallmedizin und nichteinwilligungsfähige Patient*innen verwendet. Ausgeschlossen wurden Publikationen zu Patient*innen, die psychiatrisch erkrankt sind oder im Rahmen einer Behinderung eine dauerhafte Betreuung benötigen. Es wurde anschließend eine gemeinsame Arbeitsgruppe bestehend aus Mitgliedern der Ethikkommission der Universität zu Köln, einer Juristin des Amtsgerichts Köln und Studienärzt*innen der internistischen Intensivstation der Uniklinik Köln gegründet, die in regelmäßigen Sitzungen sowohl die vorhandenen wissenschaftlichen Publikationen als auch juristische Ausführungen, Positionspapiere bzw. Stellungnahmen zu diesem Thema ausgewertet haben, um auf dieser Grundlage und mit der klinischen Erfahrung in der Durchführung von klinischen Studien in der Intensiv- und Notfallmedizin ein Modell für nichteinwilligungsfähige Patient*innen zu entwickeln.

## Ergebnisse

Die Recherche der internationalen Literatur konnte nur eingeschränkt als Informationenquelle genutzt werden [2–5]. Es gibt international teils erhebliche soziale, juristische, administrative und kulturelle Unterschiede in der Bewertung der Studienteilnahme von nichteinwilligungsfähigen Patient*innen. Es wurden zur weiteren Erstellung des Modells hauptsächlich deutschsprachige Artikel und juristische Stellungnahmen berücksichtigt [6–19].

In den ersten Sitzungen der Arbeitsgruppe wurde rasch deutlich, dass durch unterschiedliche wissenschaftliche Untersuchungsmethoden zum Teil sehr unterschiedliche ethische Aspekte und Betrachtungsweisen in die Bewertung einer Studie einfließen.

Grundsätzlich muss für klinische Studien oder andere Forschungsprojekte, die Menschen involvieren, vorab das Votum einer Ethikkommission eingeholt werden (§ 15 Abs. 1 [Muster-]Berufsordnung für die in Deutschland tätigen Ärztinnen und Ärzte – MBO‑Ä 1997 – in der Fassung der Beschlüsse des 114. Deutschen Ärztetags 2011 in Kiel); für klinische Prüfungen von Arzneimitteln oder Medizinprodukten bedarf es neben der zustimmenden Bewertung durch die Ethikkommission auch einer Genehmigung durch die zuständige Bundesoberbehörde. Bei Arzneimittelprüfungen ist die Stellungnahme der Ethikkommission für die Genehmigung durch den Mitgliedsstaat von entscheidender Bedeutung. Damit erfolgt bei allen Studien und klinischen Prüfungen im *ersten Verfahrensschritt* eine Befassung der zuständigen Ethikkommission mit dem Forschungsvorhaben. Diese leistet die Nutzen-Schaden-Abwägung sowie formale Einordnung der Studie gemäß der im Folgenden beschriebenen Klassifikation in Risikokategorien und erteilt nach Beratung ggf. ein positives Votum.

**Figure 1:**
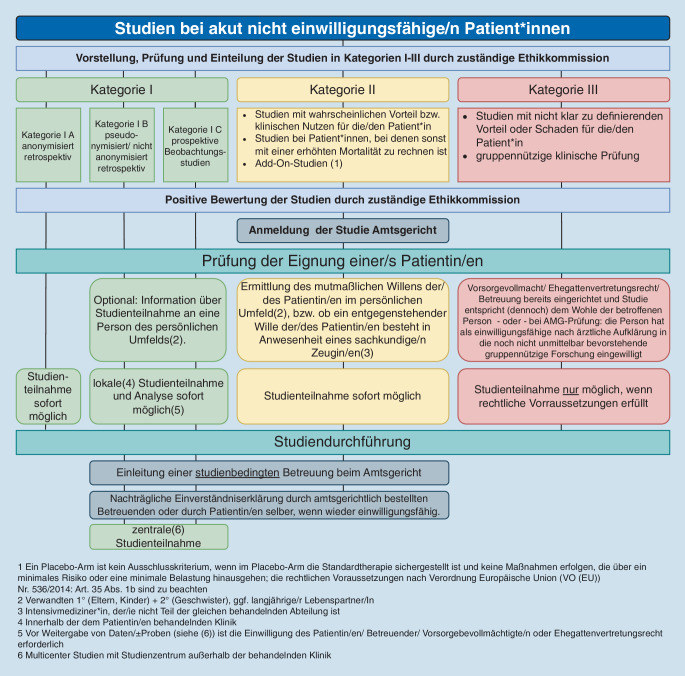

Der *zweite Verfahrensschritt *ist eine eventuelle Vorabinformation des zuständigen Amtsgerichts. Dies ist abhängig von der Art der Studie, wobei 3 Kategorien von Studien zu unterscheiden sind (siehe Abb. 1). Diese unterscheiden sich danach, ob von der klinischen Routine abgewichen wird und welcher mögliche Nutzen für die einzelnen Patient*innen (abgewogen gegen den möglichen Schaden) zu erwarten ist:

Kategorie I: Studien ohne Schadenspotenzial für das körperliche Wohlergehen der Patientin oder des Patienten

- anonymisierte retrospektive Studien
- pseudonymisierte retrospektive Studien
- prospektive Beobachtungsstudien

Kategorie II: Studien mit möglichem Eigennutzen für Patient*innen

- Studien mit möglichem klinischem Nutzen für die Patientin oder den Patienten bei geringen Belastungen und vertretbarem Schadensrisiko
- klinische Situationen, in denen mit einer hohen Mortalität zu rechnen ist, jedoch eine neue Therapieoption mit vertretbarem Sicherheitsrisiko angeboten werden kann
- Add-On-Studien. Hierbei ist die Anwendung eines Placebos kein Ausschlusskriterium, wenn im Placeboarm die Standardtherapie sichergestellt ist und keine Maßnahmen erfolgen, die über ein minimales Risiko oder eine minimale Belastung hinausgehen (die rechtlichen Voraussetzungen nach VO (EU) Nr. 536/2014: Art. 35 Abs. 1b sind zu beachten)

Kategorie III: Studien ohne möglichen Eigennutzen für Patient*innen

- Erkenntnisgewinn für die Patient*innengruppe steht im Vordergrund der Fragestellung
- nicht klar zu definierender Vorteil für die einzelnen Patient*innen

In diesem zweiten Verfahrensschritt werden *nur die Studien der Kategorie II beim zuständigen Amtsgericht vorgestellt.* Dies dient der Vorabinformation der für die Einrichtung der Betreuung zuständigen Amtsrichterin oder des Amtsrichters. Das Betreuungsgericht hat nicht über die Studie als solche zu entscheiden, könnte z. B. aber an dieser Stelle einwenden, dass die Einrichtung von Betreuungen für diese Studie fraglich erscheint, etwa weil kein möglicher Vorteil für die Patient*innen erkannt werden kann.

Denn nur bei der Kategorie II kann sich die Notwendigkeit einer Betreuungseinrichtung ergeben: Rechtlich ist grundsätzlich Voraussetzung für die Teilnahme an einer Studie, dass vor Beginn der Studie die Teilnehmerin oder der Teilnehmer bzw. seine/ihre Vertretung über die Studie aufgeklärt worden ist und einer Teilnahme an ihr zugestimmt hat. Ist dies der Fall, ist eine Involvierung des Betreuungsgerichts nicht nötig. Jedoch ist auch ohne Aufklärung/Zustimmung der Studienteilnehmer*innen/Vertretung gemäß Art. 35 Absatz 1 b) VO (EU) Nr. 536/14 eine Studienteilnahme möglich, wenn es wissenschaftliche Gründe für die Erwartung gibt, dass die Teilnahme der Prüfungsteilnehmer*innen an der klinischen Prüfung unter Umständen einen direkten klinisch relevanten Nutzen für die Prüfungsteilnehmer*innen zur Folge hat, kein entgegenstehender Wille der betroffenen Person bekannt ist und eine informierte Einwilligung der Patientin oder des Patienten oder seiner gesetzlichen Vertretung nicht möglich ist. In solchen Fällen ist die Zustimmung der gesetzlichen Vertretung unverzüglich nachzuholen. Gibt es keine*n in diesem Sinne Berechtigte*n, muss das Betreuungsgericht hierfür eine*n Betreuer*in bestellen. Durch die Vorabinformation über die Studie soll das Betreuungsverfahren beschleunigt werden, da die für die Betreuungsbestellung zu klärende Vorfrage, ob eine Teilnahme der Patientin oder des Patienten aufgrund eines zu erwartenden Vorteils möglich ist, bereits vom Betreuungsgericht geklärt werden konnte.

Der *dritte Verfahrensschritt* ist dann der Studieneinschluss von für die Studie geeigneten Patient*innen.

### Kategorie I

Mit dieser Kategorie werden Studien erfasst, in denen routinemäßig erhobene klinische Daten retrospektiv ausgewertet werden. Eine Zustimmung der Patientin/des Patienten/seines gesetzlichen Vertreters ist lediglich für die Datenverarbeitung/-weitergabe von nicht anonymisierten patient*innenspezifischen Daten an Dritte von Belang. Ein sofortiger Studieneinschluss der Patientin/des Patienten ist grundsätzlich möglich, wenn die personenbezogenen Daten zunächst nur lokal verarbeitet werden.

### Kategorie II

In einem Gespräch mit Verwandten ersten und/oder zweiten Grades oder einem/einer langjährigen Lebenspartner*in soll der mutmaßliche Wille der Patientin/des Patienten hinsichtlich einer Studienteilnahme ermittelt werden. Auch wenn von gesetzlicher Seite ein solches Gespräch nur zum Ziel haben müsste, einen entgegenstehenden Willen zu ermitteln, erscheint es aus ethischen Gründen, aber auch zur Erhaltung des Vertrauensverhältnisses erforderlich, den Willen der Patientin/des Patienten nicht nur negativ, sondern auch positiv zu ermitteln. Dies soll in Anwesenheit einer Intensivmedizinerin oder eines Intensivmediziners stattfinden, die/der nicht Teil des Behandlungsteams ist. Die Studien dieser Kategorie lassen einen Vorteil der Patient*innen erwarten, weshalb eine sofortige Studienteilnahme möglich ist, bevor die Prüfungsteilnehmerin/der Prüfungsteilnehmer bzw. ihre/seine gesetzliche Vertretung zugestimmt hat. Diese ist dann unverzüglich nachzuholen (vgl. Art. 35 Absatz 2V O [EU] Nr. 536/214). Das Besondere an diesem Vorgehen ist der unmittelbare Beginn der Studientherapie. Ein Abwarten auf die Einrichtung einer Betreuung durch das Amtsgericht ist nicht notwendig. Da die Aufklärung über die Studie und die Zustimmung zu der Teilnahme unverzüglich nachzuholen sind, muss unmittelbar nach Studienbeginn entweder die Patientin/der Patient selbst oder ihre/seine bereits vorhandene gesetzliche Vertretung aufgeklärt werden und zustimmen. Ist dies nicht möglich, weil die Patientin/der Patient auch nachträglich nicht zustimmen kann und eine rechtliche Vertretung nicht besteht, ist zu diesem Zweck eine Betreuung durch das bereits informierte Betreuungsgericht einzurichten.

### Kategorie III

Hier ist eine Studienteilnahme nur möglich, wenn hierzu eine berechtigte Vertretung der Patientin/des Patienten der Studienteilnahme zustimmt. Hierzu ist etwa eine erteilte Vorsorgevollmacht oder die Voraussetzungen des seit dem 01.01.2023 geltenden Ehegattenvertretungsrechts zu prüfen. Eine gesetzliche Betreuung hätte den (mutmaßlichen) Willen der Patientin/des Patienten zu beachten. Kritisch wird in diesen Fällen auch vom der gesetzlichen Vertretung zu prüfen sein, ob die Teilnahme dem Wohl der betreuten Person entspricht.

## Diskussion

Durch die VO (EU) Nr. 536/2014 besteht nicht nur ein Harmonisierungsbedarf beim Antrag auf Genehmigung von klinischen Prüfungen allgemein, sondern insbesondere auch bei den zuständigen Ethikkommissionen. Durch das hier aufgeführte Modell wird versucht, sowohl den rechtlichen Aspekten, dem Patient*innenschutz, als auch der klinischen Forschung in diesem neuen Umfeld gerecht zu werden. Hierdurch soll sowohl der Ethikkommission als auch den Forschenden ein Leitfaden für die Bewertung bzw. Planung von klinischen Studien an die Hand gegeben werden.

Bisher gab es schon einige Initiativen bzw. Modelle für Verfahren zur Teilnahme an intensiv- und notfallmedizinischen Studien bei nichteinwilligungsfähigen Patient*innen. Das „Gießener Modell“ beinhaltet die Einholung der Meinung einer/eines beliebigen an der klinischen Prüfung nicht beteiligten Mediziner*in [20]. Das „Heidelberger Verfahren“ sieht vor: Auch akut nichteinwilligungsfähige Patient*innen sollten nach § 41 Abs. 3 AMG a. F. unterliegen (Schutzvorschriften zugunsten Einwilligungsunfähiger und § 41 Abs. 1 AMG a. F.). Zur Not sollte eine Eilentscheidung durch einen Betreuungsrichter*in [15, 21] getroffen werden. Das „Münsteraner Modell“ sieht vor in dem besonderen Fall einer Einwilligungsunfähigkeit aufgrund eines Notfalls, dass dies geregelt wird durch das AMG in § 41 Abs. 1 S. 2 und 3. Nach Anwendung der für den jeweiligen Notfall indizierten, schulmedizinischen Behandlungsverfahren dürfen diese Patient*innen an einer klinischen Prüfung teilnehmen, wenn die Voraussetzungen (s. a. [22]) eingehalten werden. Es wird grundsätzlich keine Entscheidung einer Vertretung benötigt (sofern ein solcher nicht bereits vor dem Notfall vorhanden und im Notfall erreichbar ist). Die Ethikkommission hat hierbei eine zentrale steuernde Funktion.

In einer Publikation von den Kolleg*innen Weimann et al. über den Einschluss von Intensivpatient*innen in klinischen Studien werden die ethischen, rechtlichen und organisatorischen Probleme aus interdisziplinärer Sicht herausgearbeitet und diskutiert [23]. Hier werden die unterschiedlichen Arten von klinischen Studien gut definiert und dienen als Grundlage für unsere Einteilung. Die Autor*innen vertreten die Meinung, dass ein Studieneinschluss im Patient*inneninteresse auch dann gerechtfertigt ist, wenn die zu prüfende therapeutische Intervention für sich allein nicht lebensrettend ist, aber im therapeutischen auf Heilung ausgerichteten Gesamtkonzept günstige Effekte für die Patientin/den Patienten erwartet werden dürfen. Dies gilt sogar für Kontrollpatient*innen, da unter kontrollierten Studienbedingungen häufig eine bessere Standardbehandlung durchgeführt wird [24]. Das in der Publikation vorgeschlagene Modell des Kompetenznetzwerks Sepsis (SepNet) sieht bei nichteinwilligungsfähigen Patient*innen, die keine Betreuung haben, das unabhängige Konsilarztverfahren vor. Dieser bestätigt schriftlich die Nichteinwilligungsfähigkeit der Patientin oder des Patienten, die Dringlichkeit der Studienteilnahme sowie den möglichen Nutzen für die Patientin oder den Patienten in der Kombination mit einer Einrichtung einer Eilbetreuung. Ausführlich werden in dem Manuskript die Vorteile, aber auch Nachteile dieses Modells diskutiert. Im Gegensatz zu dem von uns vorgestellten Modell hat sich eine Eilbetreuung in vielen Amtsgerichten bzw. Betreuungsgerichten nicht realisieren lassen oder war zeitlich so verzögert, dass eine Studienteilnahme nicht mehr möglich war. Zudem hat eine zunehmende Anzahl von Ethikkommissionen nicht den möglicherweise vorhandenen Nutzen einer Studienteilnahme, sondern die Ermittlung des vermutlichen Willens der Patientin oder des Patienten in den Vordergrund gestellt.

In unserem Kölner Modell wurde versucht, klare Definitionen für unterschiedliche Studienarten (retrospektive Studien, prospektive Studien, Phase-I- bis -III-Studie bzw. gruppennützige Forschung) für die Einteilung in die Kategorien I bis III zu benennen, um so eine differenzierte Betrachtung für den Einschluss nichteinwilligungsfähiger Patient*innen zu machen. Die meisten Studien können in der Regel eindeutig zugeordnet werden. Unklare Studienarten müssen dann mit der Ethikkommission und der zuständigen Studienleitung in eine der 3 Kategorien zugeordnet werden. Hier gibt es noch keine konkreten Verfahrensanweisungen, allerdings halten wir die Anzahl dieser nicht eindeutig zuordbaren Studien für sehr gering.

In Studien der Kategorie I werden routinemäßig erhobene klinische Daten retrospektiv ausgewertet. Eine Zustimmung der Patient*innen/der gesetzlichen Vertretung ist lediglich für die Datenverarbeitung/-weitergabe von nichtanonymisierten patient*innenspezifischen Daten an Dritte vorgesehen.

Dass es unterschiedliche Rechtsauffassungen für Studien der Kategorie II und III gibt, zeigt die vorhandene Literatur zu diesem Thema. In der Beurteilung von Uni.-Prof. Dr. jur. Thomas Gutmann (Lehrstuhl für Bürgerliches Recht, Rechtsphilosophie und Medizinrecht, Universität Münster) stellt er fest, dass ein studienbedingter Einschluss von nichteinwilligungsfähigen Patient*innen möglich ist, wenn er dem mutmaßlichen Willen der Patient*innen entspricht (§ 630d Abs. 1 Satz 4 BGB). Dieser ist in der Regel über Angehörige ersten und zweiten Grades oder langjährige Lebenspartner*innen zu ermitteln.

Ob Angehörige oder die Lebenspartnerin oder der Lebenspartner in der Lage sind, eine Entscheidung über eine Studienteilnahme abzugeben, ist Gegenstand einiger internationaler Untersuchungen. In diesem Zusammenhang untersuchten Gigon et al., wie sich die Invasivität einer Studie auf die Entscheidung darüber auswirkt, wer die Zustimmung erteilen soll und wie die informierte Zustimmung erfolgen soll [2]. Bei der Entlassung von der Intensivstation wurden Patient*innen und Angehörige nach dem Zufallsprinzip ausgewählt, ob sie die Vignette einer nichtinvasiven oder einer invasiven Studie erhalten, und jede Vignette enthielt Fragen zu einer/einem bewusstlosen Patient*in und einer/einem Patient*in bei Bewusstsein. Es wurden 185 Patient*innen und 125 Familienangehörige eingeschlossen. Die Invasivität der Studie hatte keinen Einfluss auf die Entscheidung der Befragten, wer die Einwilligung geben sollte, aber die invasivere Studie veranlasste die Befragten eher dazu, mehr als eine Person in den Einwilligungsprozess einzubeziehen, und die Akzeptanz einer aufgeschobenen oder 2‑stufigen Einwilligung nahm ab. In einer weiteren Beobachtungsstudie untersuchten Potter et al. Patient*innen, die an der NICE-SUGAR-Studie („normoglycemia in intensive care and survival using glucose algorithm regulation“) teilgenommen haben, über die „verzögerte Einwilligung“, die in dieser Studie eingesetzt wurde [3]. Die NICE-SUGAR-Studie war eine multizentrische randomisierte kontrollierte Studie, die in Australien, Neuseeland und Nordamerika durchgeführt wurde. Sie untersuchten 298 der NICE-SUGAR-Teilnehmer*innen (79 % Antwortquote), von denen bei 27 % der Teilnehmer*innen eine verzögerte Einwilligung und von 72 % eine Ersatzeinwilligung eingeholt wurde. Von diesen Teilnehmern*innen hätten 96 % an NICE-SUGAR teilgenommen, wenn sie vor der Einschreibung gefragt worden wären, und 82 % hätten die Person, die tatsächlich in ihrem Namen zugestimmt hat, als erste Wahl angegeben. Die Autor*innen kamen zu dem Schluss, dass der in NICE-SUGAR angewandte Einwilligungsansatz, einschließlich der verzögerten Einwilligung, wenn keine Ersatzperson zur Verfügung stand, für die große Mehrheit der Studienteilnehmer*innen akzeptabel war. Untersuchungen aus Deutschland liegen diesbezüglich nicht vor. Es kann jedoch davon ausgegangen werden, dass die soziokulturellen Unterschiede innerhalb Europas und Australiens/Neuseelands in dieser Frage nicht sehr ausgeprägt sind und eine Übertragbarkeit der Untersuchungsergebnisse statthaft ist.

Damit sind für Studien der Kategorie II vorgesehene Befragungen der Angehörigen ersten und zweiten Grades sowie langjähriger Lebensgefährt*innen über den mutmaßlichen Willen der Patientin/des Patienten aus unserer Sicht ausreichend. Anders sieht es bei Studien der Kategorie III aus. Hier hat die Patientin/der Patient keinen Vorteil durch die Teilnahme an der Studie bzw. kann sogar Schaden davontragen. Ebenfalls gilt dies für gruppennützige Forschung, die allerdings in der Intensivmedizin selten ist.

Neu in der Bewertung ist das Ehegattenvertretungsrecht (§ 1358 BGB), das seit dem 01.01.2023 gilt. Nach bis dahin geltendem Recht konnten Ehegattin bzw. Ehegatten weder Entscheidungen über medizinische Behandlungen für ihre*n nicht mehr selbst handlungsfähige*n Partner*in treffen noch diesen im Rechtsverkehr vertreten, solange sie nicht als gesetzliche*r Betreuer*in bestellt wurden oder zuvor von der/dem Ehepartner*in bevollmächtigt wurden. In diesem neuen Gesetz ist es möglich, dass eine gegenseitige Vertretung von Ehegatten in Angelegenheiten u. a. der Gesundheitssorge besteht. Das Ehegattenvertretungsrecht gilt nicht, wenn sich die Ehepartner in Trennung befinden bzw. getrennt leben, bei Ablehnung des Vertretungsrechts durch die/den vertretene*n Ehegatten*in und wenn es eine schon offiziell bestellte Vertretung/Betreuung gibt und es keinen Ausschlussgrund für das Vertretungsrecht gibt. Mit dieser seit 2023 bestehenden Möglichkeit ergibt sich für die Durchführung von klinischen Studien im intensivmedizinischen Bereich eine deutliche Vereinfachung für den Einschluss von nichteinwilligungsfähigen Patient*innen.

Allerdings ist die aktuelle Meinung von Intensivmediziner*innen, Ethiker*innen und Jurist*innen zum Ehegattenvertretungsrecht teilweise sehr different und Gegenstand von Diskussionen. Hauptsächlich geht es um die Frage, ob das Ehegattenvertretungsrecht auch für den Fall einer Studie greift oder ob hier eine neue Situation vorliegt. Explizit findet die Teilnahme an einer Studie und die Zustimmung durch das Ehegattenvertretungsrecht keine Erwähnung [25]. Ethiker*innen und Medizinrechtler*innen sehen hier eine mögliche Überforderung der Ehepartnerin/des Ehepartners, in einer besonders emotional belastenden Situation eine Entscheidung über eine Studienteilnahme zu treffen. Zuständige Amtsgerichte sehen unterschiedliche Ermessensbereiche und mitunter keinen Unterschied zwischen einem Behandlungsvertrag (wie es im Gesetz steht) und einem z. B. Studien(einschluss‑)Vertrag und lassen demnach die letztendliche Entscheidung offen oder aber wenden in den meisten Fällen das Ehegattenvertretungsrecht an. Zumal bislang in vielen Fällen in der Vergangenheit der Ehepartnerin/dem Ehepartner auch die medizinische Betreuung zugesprochen wurde.

Bei einwilligungsunfähigen Patient*innen ist gemäß § 630d Abs. 1 S. 2 BGB die Einwilligung eines hierzu Berechtigten einzuholen, soweit nicht eine Patient*innenverfügung nach § 1827 Abs. 1, S. 1 BGB die Maßnahme gestattet oder untersagt. Bei Vorliegen der Voraussetzungen des Ehegattennotvertretungsrechts wird der vertretende Ehegatte oder Ehegattin in diesem Sinne zum Berechtigten, der die Einwilligung erteilen kann. Dieser ist allerdings auch dann, wenn keine Patient*innenverfügung vorliegt, an den (mutmaßlichen) Willen des Vertretenen gebunden. Dies dürfte im Regelfall in den Fällen der Kategorie I/II gegeben sein; in der Kategorie III ist dies unwahrscheinlich, aber nicht von vornherein ausgeschlossen. In jedem Fall beachtlich ist § 1829 BGB soweit die begründete Gefahr besteht, dass der Patient durch die ärztliche Maßnahme stirbt oder einen schweren und länger dauernden gesundheitlichen Schaden erleidet.

Ein weiterer wichtiger Punkt ist aus unserer Sicht das enge zeitliche Fenster für einen Studieneinschluss der Patientin/des Patienten. Je nach Studie in Kategorie II beträgt dieses nur wenige Stunden. Je nach Tageszeit und Wochentag oder an Feiertagen bedeutet dies einen erheblichen organisatorischen Aufwand. Ebenfalls gibt es erhebliche Unterschiede in der Bearbeitungsdauer bzw. Schnelligkeit an deutschen Betreuungsgerichten. Durch die Vorabinformation und der „nur“ studienbedingten Betreuungseinrichtung kann der administrative Aufwand deutlich verringert werden, da zusätzliche Aspekte des sonst üblichen Betreuungsverfahrens ausgeblendet werden können. Sowohl im Ehegattenvertretungsrecht als auch in der studienbedingten Betreuungseinrichtung sind die Berechtigungen des/des vertretenden Ehepartner*in bzw. der dann bestellten Betreuungsperson u. a. nur auf medizinische Einwilligungserklärung und Behandlungsverträge (§§ 630a ff BGB und §§ 611 ff BGB i.V.m, SGB V und LandesKKH‑G sowie SGB IX und SGB XI) beschränkt.

Was ist anders im Vergleich zu den Modellen aus Gießen, Heidelberg und Münster? In dem neuen Kölner Modell wurden die neuen europäischen Verordnungen ([EU] Nr. 536/2014) und das Ehegattennotvertretungsrecht aufgenommen. Zum anderen wurde bewusst Wert darauf gelegt, dass nicht alle Studien gleich sind und eine differenzierte Bewertung in unterschiedliche Kategorien im Vorfeld stattfinden muss. Anonymisierte retrospektive Datenanalysen haben sicherlich einen anderen Stellenwert wie randomisierte placebokontrollierte Studien.

Ein bundesweiter Einsatz eines einheitlichen Modells wäre wünschenswert, um eine Harmonisierung in der Durchführung von Studien bei nichteinwilligungsfähigen Patientinnen und Patienten zu bekommen. Insgesamt sehen wir mit dem hier vorgestellten Kölner Modell eine gute Möglichkeit im Zusammenhang mit klinischen Studien, die notwendigen Schutzmaßnahmen für gefährdete Patient*innen bei gleichzeitig praktikabler juristischer Umsetzung zu beachten und umzusetzen. Damit kann eine zeitnahe und effiziente Forschung durchgeführt werden, die valide und verallgemeinerbar ist, um die Ergebnisse für alle kritisch kranken Patient*innen und ihre Familien in Deutschland zu verbessern. Auch durch die neuen Erfordernisse der EU-Verordnung 536/2014 steht mit dem Kölner Modell ein Verfahren zur Verfügung, das Eingang in die tägliche Praxis finden könnte.

## References

[1] EU-Verordnung 536/2014 – AKEK – Arbeitskreis Medizinischer Ethik-Kommissionen. https://www.akek.de/aktuelle-hinweise/eu-verordnung-536-2014/. Zugegriffen: 20. Jan. 2023

[2] Gigon F, Merlani P, Chenaud C, Ricou B (2013). ICU research: the impact of invasiveness on informed consent. Intensive Care Med..

[3] Potter JE, McKinley S, Delaney A (2013). Research participants’ opinions of delayed consent for a randomised controlled trial of glucose control in intensive care. Intensive Care Med..

[4] Chenaud C, Merlani P, Verdon M, Ricou B (2009). Who should consent for research in adult intensive care? Preferences of patients and their relatives: a pilot study. J Med Ethics.

[5] Dahlberg J, Eriksen C, Robertsen A, Beitland S (2020). Barriers and challenges in the process of including critically ill patients in clinical studies. Scand J Trauma Resusc Emerg Med.

[6] Dick AFW, Encke A, Schuster HP (1996). Forschung und Ethik in der Notfallmedizin Empfehlungen eines Workshops. Anaesthesist.

[7] Hansen H-C, Drews R, Gaidzik PW (2008). Zwischen Patientenautonomie und ärztlicher Garantenstellung. Nervenarzt.

[8] Prüß H, Köhler S, Müller S (2020). Autoimmune Enzephalitiden – diagnostischer und therapeutischer Entscheidungsbaum aus psychiatrischer, neurologischer und ethisch-juristischer Sicht. Nervenarzt.

[9] Biermann E (2019). Einwilligung nach Aufklärung – ein juristisches Update. Anästhesiol Intensivmed Notfallmed Schmerzther.

[10] Neubauer H, Wetterling T, Neubauer W (1994). Einwilligungsfähigkeit bei älteren, vor allem dementen und verwirrten (deliranten) Patienten. Fortschr Neurol Psychiatr.

[11] Klein SJ, Voithofer C, Ganner M (2021). Nicht einwilligungsfähige Patienten in der Intensiv- und Notfallmedizin. Wien Klin Mag.

[12] Bundesärztekammer (2019). Hinweise und Empfehlungen zum Umgang mit Vorsorgevollmachten und Patientenverfügungen im ärztlichen Alltag. Jahrb Wiss Ethik.

[13] Pramann O (2017). Einwilligung des Patienten. Rechtliche Details, die Ärzte kennen sollten. Dtsch Ärztebl.

[14] Rittner C (2007). Ein Modell für die Forschung am einwilligungsunfähigen (bewusstlosen) Notfallpatienten. MedR.

[15] Steiner T, Walter-Sack I, Taupitz J (2008). Ethische und juristische Aspekte beim Einschluss nicht einwilligungsfähiger Patienten in Akuttherapie-Studien. Dtsch Med Wochenschr.

[16] Einschluss von Notfallpatienten in klinischen Studien. https://www.medizin.uni-muenster.de/ek/ethik-kommission/rechtliche-sonderthemen/nicht-einwilligungsfaehige/einschluss-von-notfallpatienten-in-klinischen-studien.html. Zugegriffen: 23. Febr. 2022

[17] Studien an akut nicht-einwilligungsfähigen Patienten … aus juristischer Sicht. https://www.akek.de/wp-content/uploads/Vortrag-Gutmann.pdf. Zugegriffen: 19. Jan. 2023

[18] Ehegattennotvertretung BÄK. https://www.bundesaerztekammer.de/service/muster-formulare. Zugegriffen: 19. Jan. 2023

[19] Gesetz zur Reform des Vormundschafts- und Betreuungsrechts. https://www.bmj.de/SharedDocs/Gesetzgebungsverfahren/DE/Reform_Betreuungsrecht_Vormundschaft.html. Zugegriffen: 19. Jan. 2023

[20] Habermann (2000). Therapeutische Prüfungen an Nicht-Einwilligungsfähigen im Eilfall – ethisch geboten und rechtlich zulässig?.

[21] Brückner UB, Brockmeyer NH, Gödicke P (2010). Einbeziehung von volljährigen einwilligungsunfähigen Notfallpatienten in Arzneimittelstudien. MedR.

[22] https://www.medizin.uni-muenster.de/ek/ethik-kommission/rechtliche-sonderthemen/nicht-einwilligungsfaehige/einschluss-von-notfallpatienten-in-klinischen-studien.html. Zugegriffen: 28.6.2023

[23] Weimann A, Kern BR, Löffler M (2013). Der Einschluss von Intensivpatienten in klinische Studien. Med Klin Intensivmed Notfallmed.

[24] Simpson SH, Eurich DT, Majumdar SR (2006). A meta-analysis of the association between adherence to drug therapy and mortality. BMJ.

[25] https://www.gesetze-im-internet.de/bgb/__1358.html. Zugegriffen: 25.7.2023

